# Immunomodulatory Protein from *Ganoderma microsporum* Induces Pro-Death Autophagy through Akt-mTOR-p70S6K Pathway Inhibition in Multidrug Resistant Lung Cancer Cells

**DOI:** 10.1371/journal.pone.0125774

**Published:** 2015-05-06

**Authors:** Ling-Yen Chiu, Ming-E Hu, Tsung-Ying Yang, I-Lun Hsin, Jiunn-Liang Ko, Kan-Jen Tsai, Gwo-Tarng Sheu

**Affiliations:** 1 Institute of Medicine, Chung Shan Medical University, Taichung, Taiwan; 2 Department of Anatomy, School of Medicine, Chung Shan Medical University, Taichung, Taiwan; 3 Division of Chest Medicine, Department of Internal Medicine, Taichung Veterans General Hospital, Taichung, Taiwan; 4 Department of Medical Oncology and Chest Medicine, Chung Shan Medical University Hospital, Taichung, Taiwan; 5 School of Medical Laboratory and Biotechnology, Chung Shan Medical University, Taichung, Taiwan; School of Medicine, University of Belgrade, SERBIA

## Abstract

Chemoresistance in cancer therapy is an unfavorable prognostic factor in non-small cell lung cancer (NSCLC). Elevation of intracellular calcium level in multidrug resistant (MDR) sublines leads to sensitization of MDR sublines to cell death. We demonstrated that a fungal protein from *Ganoderma microsporum*, GMI, elevates the intracellular calcium level and reduces the growth of MDR subline via autophagy and apoptosis, regardless of p-glycoprotein (P-gp) overexpression, in mice xenograft tumors. In addition, we examined the roles of autophagy in the death of MDR A549 lung cancer sublines by GMI, thapsigargin (TG) and tunicamycin (TM) *in vitro*. Cytotoxicity of TG was inhibited by overexpressed P-gp. However, TM-induced death of MDR sublines was independent of P-gp level. Combinations of TG and TM with either docetaxel or vincristine showed no additional cytotoxic effects on MDR sublines. TG- and TM-mediated apoptosis of MDR sublines was demonstrated on Annexin-V assay and Western blot and repressed by pan-caspase inhibitor (Z-VAD-FMK). Treatment of MDR sublines with TG and TM also augmented autophagy with accumulation of LC3-II proteins, breakdown of p62 and formation of acidic vesicular organelles (AVOs). Inhibition of ATG5 by shRNA silencing significantly reduced autophagy and cell death but not apoptosis following TG or TM treatment. GMI treatment inhibited the phosphorylation of Akt/S473 and p70S6K/T389. Interestingly, the phosphorylation of ERK was not associated with GMI-induced autophagy. We conclude that autophagy plays a pro-death role in acquired MDR and upregulation of autophagy by GMI via Akt/mTOR inhibition provides a potential strategy for overcoming MDR in the treatment of lung cancers.

## Introduction

Lung cancer is the most common malignancy among men and women. Although chemotherapy is the suggested treatment for advanced non-small cell lung cancer (NSCLC), most patients develop cross-resistance to a diversity of chemotherapeutic drugs (multiple drug resistance, MDR) [1]. P-glycoprotein (P-gp), the product of the human *MDR-1* gene, is a member of the large ATP-binding cassette family of membrane proteins [2]. P-gp, if overexpressed in cancer cells, can pump anticancer drugs out of the cells. MDR is a critical problem in chemotherapy. Novel drugs and strategies are needed to overcome MDR to obtain better prognoses. To improve the efficacy of chemotherapy, use of Chinese herbal medicines as MDR reversing agents [3], MDR modulators of ATP-binding cassette transporters [4] and nanomedical solutions for circumventing MDR has been discussed extensively [5].

Both docetaxel (DOC) [6] and vincristine (VCR) [7] are tubulin binding agents (TBA) that have been applied clinically to various cancer chemotherapy regimens. However, they are capable of inducing MDR. We used DOC and VCR as selection agents to treat A549 NSCLC cells. Under continuous exposure, several DOC and VCR drug resistant sublines were obtained for further investigation. In a previous study, we reported that when combined with DOC or VCR, three L-type calcium channel blockers, verapamil (VER), nifedipine and diltiazem, reverse MDR with different efficacies in DOC- and VCR-resistant sublines regardless of the expression levels of efflux transporters [8]. Therefore, it is logical to assume that calcium channel blocker activity is associated with reversal of MDR ability. Accumulated data have indicated that TBA treatment leads to a disruption of calcium homeostasis [9]. Thus, low level intracellular calcium pool may permit MDR-positive cells to sustain free radical-induced damage in association with other unidentified factors. Taken together, alteration of intracellular calcium level ([Ca^2+^]_cyt_) in TBA-resistant lung cancer sublines may modulate chemoresistance and ([Ca^2+^]_cyt_)-mediated pathways are potential targets for overcoming MDR.

The endoplasmic reticulum (ER) is an intracellular calcium storage partition that plays a role in the preservation of cellular calcium homeostasis [10]. Perturbations in ER homeostasis affect protein folding to generate the unfold protein response (UPR), also called ER stress [11]. ER stress can be secured by pharmacological agents including thapsigargin (TG) [12] and tunicamycin (TM) [13]. When cells are treated with TG, [Ca^2+^]_cyt_ increases [14] to generate autophagy [15] and apoptosis [16]. Treatment with TM leads to induction of ER stress with increased [Ca^2+^]_cyt_ and is also associated with apoptosis [17] and autophagy [15,16]. Three ER stressors, TM, dithiothreitol (DTT) and proteasome inhibitor MG132, have been tested in mouse embryo fibroblasts (MEFs) and found to induce autophagy by negatively regulating Akt/mammalian target of rapamycin (mTOR) pathway [18]. However, clear evidence of a mechanism by which autophagy regulates cell death in MDR cancers is still lacking.

Programmed cell death is classified as apoptosis, necrosis or autophagy [19,20]. Apoptotic pathways include those that are extrinsic and those that are intrinsic. Each pathway converges on aspartate-specific cysteine proteases known as caspases (initiating 8, 9, 10 and executioner 3, 6, 7) [20]. These caspases cleave and activate downstream apoptotic proteins, thereby regulating cell death. Apoptosis is a major effect of anti-cancer drug treatment. Autophagy has also been extensively studied in cancer therapy. Autophagy is controlled by a group of evolutionarily conserved autophagy gene-related proteins (ATG proteins) in a process that includes: (i) induction or initiation that correlates with formation of the phagophore; (ii) nucleation; (iii) elongation, Atg12-Atg5 and LC3/Atg8 controlled critical step in forming the autophagosomes; and (iv) maturation and degradation, in which the autophagosomes fuse with endosomes-lysosomes to form autolysosomes with degradation of the lumenal contents [21]. The appropriate level of autophagy that encourages tumor cell survival has been discussed as part of the pro-survival role of autophagy in established tumors [22,23]. Autophagy has been suggested as a target for inducing cancer cell death [24,25] and inhibition of autophagy has been found to improve outcomes in cancer therapy [26]. However, persistent stress can promote extensive autophagy, leading to cell death. Therefore, both autophagic inhibition [27] and induction have been considered in therapeutic strategies [24] and have been applied to anti-cancer treatments. Whether autophagy plays a pro-death or pro-survival role following chemotherapy-induced resistance remains unclear. Further investigation is needed to resolve this issue and to better manage acquired chemoresistance.

Recombinant fungal immunomodulatory protein, GMI, cloned from *Ganoderma microsporum* and purified, has been shown to exhibit an inhibitory effect on EGF-induced migration and invasion [28]. This protein downregulates tumor necrosis factor alpha-induced expression of matrix metalloproteinase 9 via NF-kappaB pathway [29] and induces autophagy through a calcium-mediated signaling pathway in human lung cancer A549 cells [30]. Oral administration of GMI has been demonstrated to significantly inhibit tumor growth and induce autophagy in nude mice with xenografted tumor of A549 cells [30]. Moreover, autophagosome accumulation has been shown to induce autophagic cell death via Akt/mTOR pathway in a GMI-treated lung cancer cell model [31]. To further test the antitumor function of GMI in chemoresistance, xenografted animal tumors of docetaxel-resistant A549 subline were treated with GMI and analyzed. We further characterized the roles of apoptosis and autophagy in MDR by comparing the effects of two classical ER stress inducers, TG and TM, with GMI in docetaxel- and vincristine-selected A549 lung cancer sublines.

In this study, we demonstrated the effects and limitations of autophagy on the death of MDR sublines with ER stressors, TG and TM, as well as a novel stressor, GMI, to better understand the roles of apoptosis and autophagy in anti-MDR cancer therapy. GMI-induced Akt/mTOR pathway inhibition in autophagy-associated cell death is proposed as a method for circumventing MDR.

## Materials and Methods

### Drugs and chemicals

DOC was obtained from Aventis Pharmaceuticals Inc. (Bridgewater, NJ). VCR, VER, chloroquine and TM were obtained from Sigma-Aldrich (St. Louis, MO). Z-VAD-FMK was purchased from Bachem (Torrance, CA). TG was purchased from Tocris Bioscience (Bristol, United Kingdom) and U0126 was purchased from Cell Signaling (Danvers, MA). GMI, manufactured by Mycomagic Biotechnology Co., Ltd. (Taipei, Taiwan), was generated and ameliorated from *Ganoderma microsporum* [28].

### Flow-3AM assay

Approximately 2×10^4^ cells per well were seeded onto 24-well plates. After 24 h incubation, the cells were exposed to VER in fresh medium for 2 h. Following this, cells were exposed to DOC, VCR or GMI. At the end of the exposure period, cells were trypsinized and incubated for 30 min in Hank’s buffered salt solution (HBSS) with 3 μM Flow-3AM (Invitrogen). After staining, cells were washed with HBSS twice, placed in HBSS containing 5% FBS, and analyzed by flow cytometry.

### Cell lines and cytotoxicity assay (MTT assay)

Human adenocarcinoma A549 cells were maintained as previously described [8]. The DOC and VCR resistant sublines were established from parental cells in a stepwise manner by exposure to increasing concentrations of DOC or VCR. Briefly, for DOC subline, A549 cells in low cellular density were seeded onto 10-cm Petri dish and treated with 0.5 nM DOC until the surviving cells grew to an obvious colony. The selected colony was amplified in the presence of 0.5 nM DOC until confluence before the drug dose was increased in multiples of two for the next round of selection. The DOC resistant subline maintained at 16 nM DOC was denoted A549/D16. A similar designation, A549/V16, was given to a VCR stably resistant subline. The cells were exposed to various concentrations of DOC, VCR or GMI in fresh medium for 48 h. Drug sensitivities to DOC, VCR and GMI were determined on MTT colorimetric assay. The detailed steps of MTT assay have been previously described [8]. Mean values were calculated from three independent experiments.

### Western blot analysis

The relevant procedures have been described in a previous report [8]. Proteins were reacted with one of the following: anti-LC3A (#4599), anti-LC3B (#3868), anti-PARP (#5625), anti-GADD153 (#2895), anti-GRP78 (#3177), anti-t-Akt (#9272), anti-p-Akt Ser473 (#9271), anti-p-ERK (#4370), anti-p-p70S6K Thr389 (#9234) purchased from Cell Signaling (Danvers, MA), anti-p62 (GTX100685) obtained from GeneTex (Irvine, CA) or monoclonal anti-β-actin (Sigma, AC-40). Immunohistochemistry (IHC) was carried out with polyclonal anti P-gp, followed by anti-goat IgG (Santa Cruz Biotechnology) conjugated to horseradish peroxidase. Hematoxylin was used for counterstaining.

### Annexin V assay

Cells were trypsinized and incubated for 30 min in binding buffer with propidium iodide (PI) and Annexin V (FITC Annexin V Apoptosis Detection Kit 1, BD Biosciences, San Jose, CA), followed by analysis with flow cytometry.

### Measurement of mitochondrial membrane potential by JC-1 Assay

We used JC-1 (5’,6,6’-tetrachloro-1,1’,3,3’-tetraethylbenzimidazolylcarbocyanine iodide) and a mitochondrial membrane potential disrupter, CCCP (carbonyl cyanide 3-chlorophenylhydrazone), for the study of mitochondrial membrane potential (JC-1; Molecular Probes, Eugene, OR). Polarized mitochondria appear as red and depolarized mitochondria as green. Apoptosis was indicated by an increase in the green/red fluorescence intensity ratio. For each sample, cells (5×10^5^ cells/ml) were suspended in 1 ml of PBS buffer and JC-1 (final concentration, 2.5 μg/ml) was added to the sample, which was incubated for 10 min at 37°C. The stained cells were then centrifuged at 400×g for 5 min at room temperature and the supernatant was removed carefully. The pellet was then washed with 1–2 ml of PBS. The cells were analyzed immediately with flow cytometer (BD FACSCalibur).

### Detection and quantification of acidic vesicular organelles (AVOs)

The detailed steps of AVO analysis have been previously described [30]. Briefly, after treatment, cells were washed with HBSS twice, followed by staining with 1 g/ml acridine orange (Sigma, A 6014) and dilution in HBSS containing 5% FBS for 15 min. After staining, cells were washed with HBSS and suspended in HBSS containing 5% FBS. The cells were observed under a red filter fluorescence microscope. To quantify AVO formation, acridine orange stained cells were harvested and washed twice with HBSS, resuspended in HBSS containing 5% FBS and analyzed with flow cytometry and CellQuest software.

### ATG5 silencing by shRNA expressing lentivirus

Lentiviral infection of A549/D16 and A549/V16 sublines was carried out to stably integrate and express short hairpin RNA (shRNA) targeting ATG5 mRNA sequences. The detailed steps of lentivirus production have been previously described [30].

### Animal model of xenograft tumors

For *in vivo* tumor growth assay, 4-week-old male immunodeficient mice (NOD.CB17-Prkdcscid/IcrCrlBltw) were purchased from BioLASCO Taiwan Co., Ltd. (Taipei, Taiwan). Experimental procedures and handling were conducted in accordance with international guidelines for laboratory animal studies. The current study was approved by the Chung Shan Medical University Animal Care Committee (Permit Number: 1238) and all efforts were made to minimize suffering. To establish A549 tumor xenografts, mice were injected subcutaneously with 5 x 10^6^ A549/D16 cells (100 μl) plus 100 μl Matrigel (BD Biosciences, 354234). Six animals were then randomly divided into two groups consisting of three animals each. Seven days after cell implantation, mice in the PBS group were treated with 100 μl PBS by gavage once daily and served as controls. The GMI group was administered GMI (160 μg per mouse diluted in 100 μl PBS) by gavage once daily. Tumor sizes were measured every 3 days beginning on the 16th day following cell injection and tumor volume was calculated by the formula: 0.5 x larger diameter (mm) x small diameter^2^ (mm). Due to tumor size variability and small size of groups, a non-parametric Mann-Whitney U test was applied with p<0.05 was considered statistically significant. After animals were sacrificed, the tumor weights were measured on microbalance and the tumor lysates (10–50 μg) were analyzed on Western blots using Tissue Protein Extraction Buffer (FIVEphoton Biochemicals, San Diego, CA) for protein preparation.

### Statistical analysis

All values are presented as mean ± SD. Data were compared between groups using t-test and *p<0.05 was considered statistically significant.

## Results

### Low intracellular calcium levels ([Ca^2+^]_cyt_) of chemoresistant A549 cells are upregulated by combination TBA and verapamil treatment or GMI

**Fig 1:**
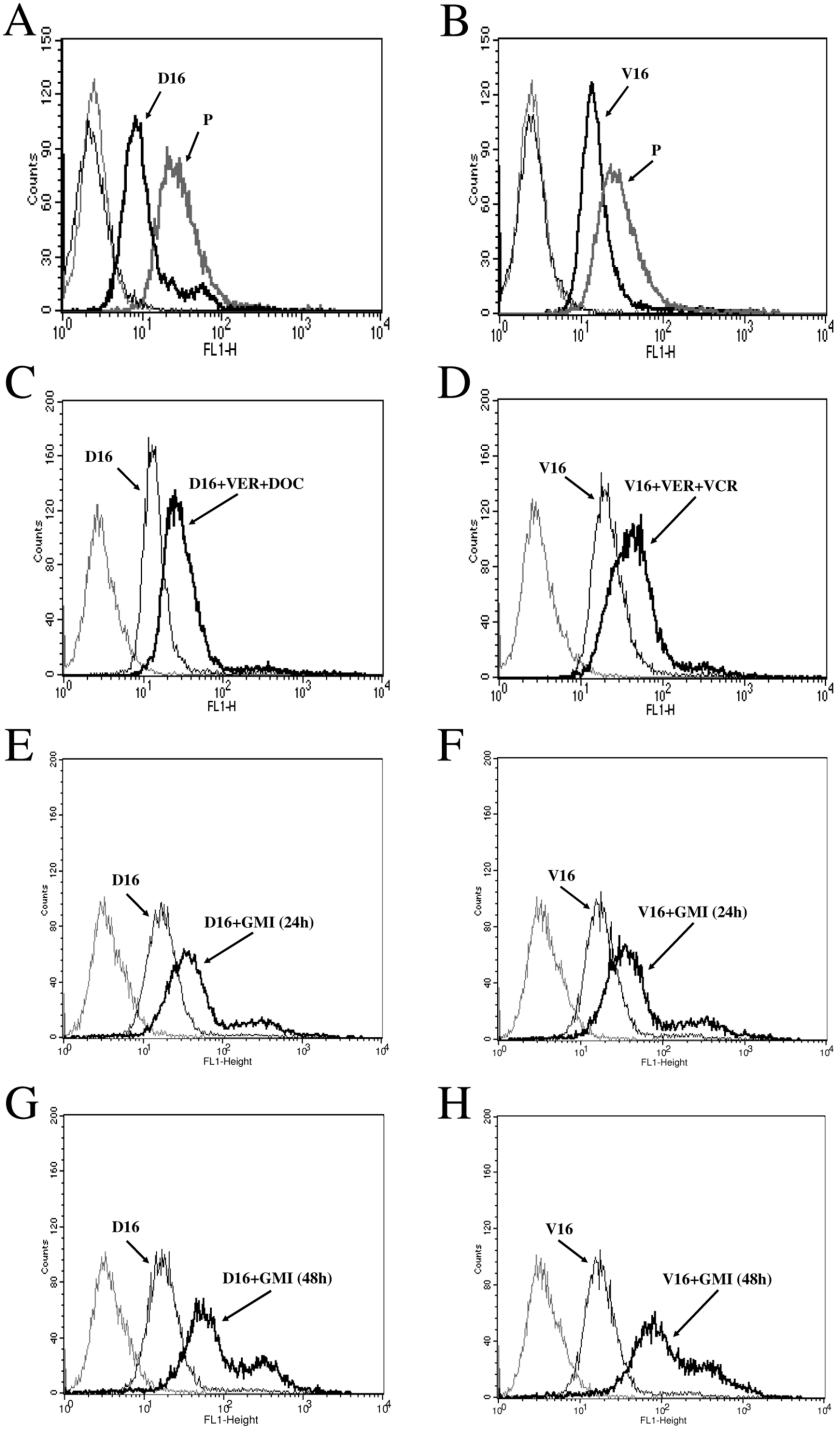
Low intracellular calcium levels [Ca^2+^]_cyt_ in chemoresistant lung cancer cells are elevated by GMI. To examine the [Ca^2+^]_cyt_, the fluorescent dye Fluo-3AM was incubated with (A) parental A549 and A549/D16 cells, (B) parental A549 and A549/V16 cells for 30 min. Thin line represents fluorescence background without Fluo-3AM incubation. Thick line represents fluorescence measured by flow cytometry. To measure VER modulation of [Ca^2+^]_cyt_, (C) A549/D16 cells and (D) A549/V16 cells were pre-treated with VER (10 μM) for 2 h followed by DOC (16 nM) and VCR (16 nM) treatment for 48 h, respectively. The [Ca^2+^]_cyt_ of A549/D16 cells treated with GMI (1.2 μM) for 24 h (E) or 48 h (G) were upregulated. The [Ca^2+^]_cyt_ of A549/V16 cells treated with GMI (1.2 μM) for 24 h (F) or 48 h (H) were also upregulated. After fluo-3 AM incubation, cells were visualized using flow cytometry. The cell number count (Count) is indicated by the Y-axis and the Fluo-3AM intensity (FL1-H) is indicated by the X-axis.

Previously, we have shown that MDR of A549/D16 subline is mediated by ABC transporters (typical MDR) and MDR of A549/V16 subline is mediated by non-ABC transporter-associated factors (atypical MDR) [8]. P-gp is upregulated in A549/D16 subline and downregulated in A549/V16 subline. Both sublines are cross-resistant to DOC and VCR. We hypothesized that the mechanism of the L-type calcium channel blockers in reversing TBA sensitivity of drug resistant cells is associated with the alteration of intracellular calcium homeostasis. To further determine the intracellular calcium levels ([Ca^2+^]_cyt_) of parental cancer cells and multidrug-resistant sublines, Fluo-3AM served as the fluorescent indicator of intracellular calcium on flow cytometry. Moreover, the alteration of [Ca^2+^]_cyt_ following combined VER and TBA treatment was determined. The results showed that [Ca^2+^]_cyt_ of multidrug-resistant, P-gp overexpressing, A549/D16 subline (Fig 1A) is maintained as demonstrated by a lower fluorescence level than parental A549 cells. Interestingly, in chemoresistant and P-gp downregulated A549/V16 subline lower [Ca^2+^]_cyt_ was also maintained (Fig 1B). The data showed that [Ca^2+^]_cyt_ in chemoresistant sublines is lower than in parental A549 cells. When the A549/D16 (Fig 1C) and the A549/V16 (Fig 1D) cells were pretreated with VER followed by TBA, the level of detected fluorescence significantly increased, implying that the [Ca^2+^]_cyt_ of chemoresistant sublines is elevated when VER re-sensitizing chemoresistant cells are exposed to TBA cytotoxicity. Upregulation of [Ca^2+^]_cyt_ by combined VER and TBA treatments was independent of the level of P-gp expression. To test the effect of GMI on [Ca^2+^]_cyt_, both sublines were treated with GMI (1.2 μM) for 24 h or 48 h followed by analysis on Fluo-3AM assay. The fluorescence signal in GMI-treated A549/D16 subline increased after 24 h (Fig 1E) and further increased after 48 h of GMI treatment (Fig 1G) Similar results were observed in A549/V16 subline treated with GMI for 24 h (Fig 1F) and 48 h (Fig 1H). The data supported our hypothesis that elevated [Ca^2+^]_cyt_ plays a significant role in chemoresistant lung cancer cell sensitization to death. Furthermore, GMI is able to induce [Ca^2+^]_cyt_ elevation in MDR sublines.

### GMI inhibits P-gp overexpressing tumor growth in mice xenograft model

**Fig 2:**
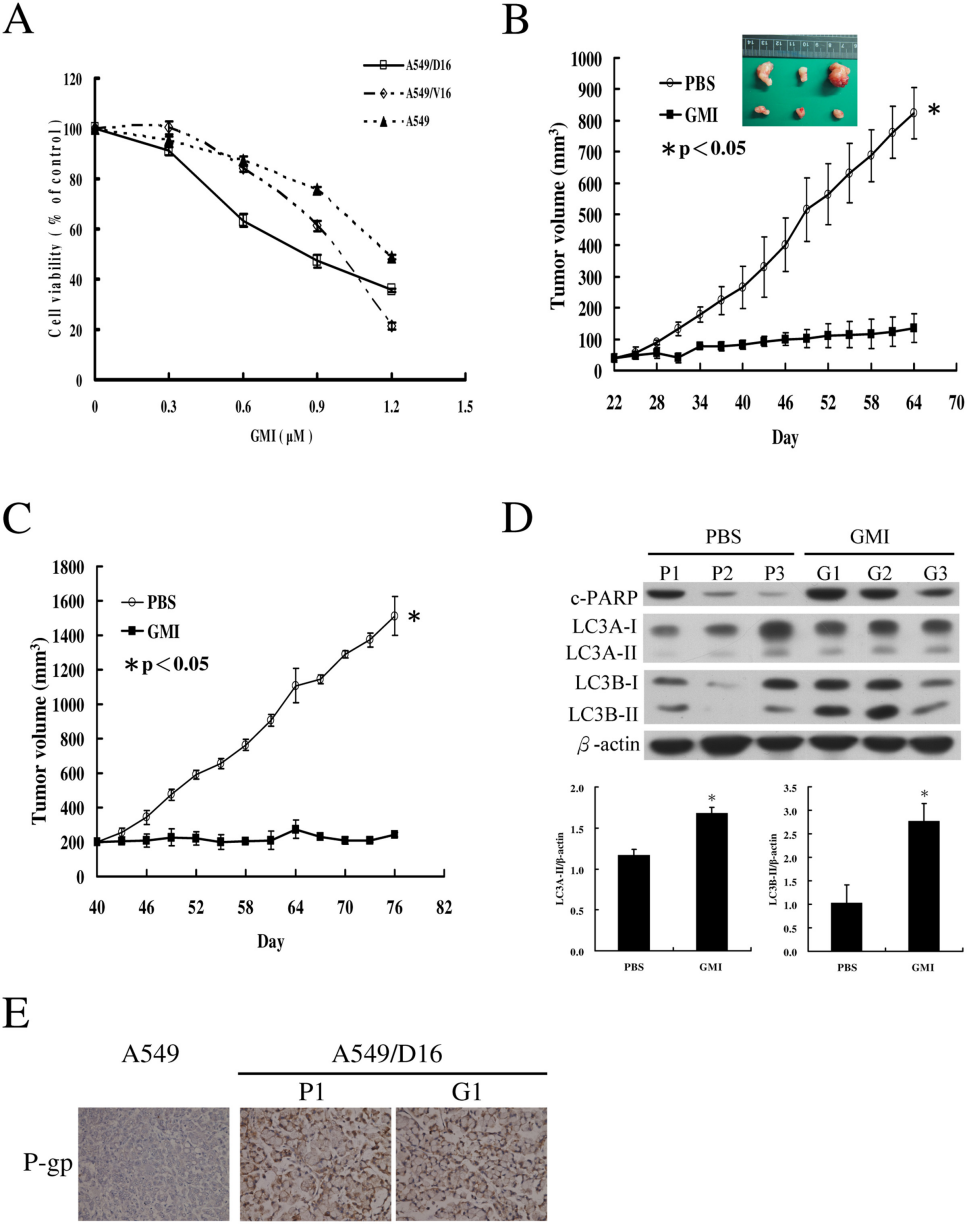
Inhibition of tumor growth by GMI in mice xenograft model. (A) Analysis of the effects of GMI (0.3, 0.6, 0.9 and 1.2 μM) on cell viability in A549, A549/D16 and A549/V16 cells on MTT assay. (B) Seven days after A549/D16 cell implantation, mice in the PBS group were treated with PBS or GMI by gavage once daily. The tumor sizes of PBS group and GMI group were measured after mice were sacrificed on day 64. (C) Mice were injected subcutaneously for A549/D16 cell implantation. When the tumor volume reached 200 mm^3^ on day 40, mice in the PBS group were treated with PBS or GMI by gavage once daily. The results are presented as mean ± SD. a non-parametric Mann-Whitney U test was applied with p<0.05 was considered statistically significant. (E) The levels of c-PARP, LC3A and LC3B in six xenograft tumors were determined by Western blotting with statistical analysis of LC3A-II and LC3B-II expressions. (D) Representative immunostaining results of P-gp in lung tumors of A549, A549/D16 treated with PBS (P1) and A549/D16 treated with GMI (G1) were obtained by IHC.

Previously, it has been demonstrated that after pretreatment with the calcium chelator BAPTA-AM, GMI-induced cell death is blocked. Apparently, GMI induces lung cancer cell death through a calcium-dependent signaling pathway [30]. Therefore, we tested the sensitivity of chemoresistant cancer cells to GMI treatment on MTT assay (Fig 2A). The results showed that both A549/D16 and A549/V16 sublines respond to GMI (0.3 to 1.2 μM) in a dose-dependent manner. The GMI sensitivities of A549, A549/D16 and A549/V16 cells with calculated IC_50_ are listed in Table 1. Moreover, mice were inoculated subcutaneously with A549/D16 subline expressing high P-gp. The tumor burdens in mice treated with PBS or GMI are shown in Fig 2B. When compared with the group of mice administered PBS, the growth of tumors in the GMI group was significantly inhibited. Although calculated tumor volume was comparable to P1 and P3, significant necrosis of P2 tumor resulted in the smallest size of the retrieved P2 tumor. To ensure proliferation of implanted cancer cells, we tested the GMI efficacy when the tumors reached a volume of 200 mm^3^ on the 40th day after implantation (Fig 2C). The anti-tumor effect of GMI was not restricted in large tumors.

**Table 1:** Drug sensitivities of parental cell line and drug resistant sublines.

Drug sensitivities of parental cell line and the drug resistant sublines			
	IC_50_ ± SD *		
Drug	A549	A549/D16	A549/V16
Thapsigargin (nM)	62.13 ± 5.44	>200	42.93 ± 1.61
Tunicamycin (μM)	0.43 ± 0.06	1.08 ± 0.14	6.20 ± 0.26
GMI (μg/ml)	15.73 ± 0.42	11.37 ± 0.55	13.10 ± 0.17

**NOTE: Cell survival was determined on MTT assay**.

On MTT toxicity assay, the drug sensitivities of established A549, A549/D16 and A549/V16 cells to TG, TM and GMI in terms of IC_50_ (Inhibition concentration) were determined.

*Data are the mean ± SD of at least three independent experiments done in triplicate.

Apoptosis-mediated cleavage of poly (ADP-ribose) polymerase (PARP-1) by capase-3 produced an 89 kDa C-terminal fragment (c-PARP, containing the catalytic domain) which served as the molecular marker on immunoblots. To elucidate the ER stress-regulated autophagy, the microtubule-associated protein light chain (LC3) protein was analyzed. LC3 has three isoforms (LC3A, LC3B, and LC3C) [32] of which LC3A and LC3B were examined. The GMI-treated tumors expressed high levels of c-PARP, LC3A-II and LC3B-II proteins, indicating higher autophagic and apoptotic activities than in the PBS-treated control tumors (Fig 2D). To demonstrate high P-gp expression in the xenograft tumors, IHC of P-gp was applied to the representative tumors of P1, G1 and parental A549 cells (Fig 2E). The P1 and G1 tumors showed strong P-gp expression in contrast to the tumors of A549 cells. The data from these mice xenograft tumor experiments demonstrated that GMI inhibits the growth of P-gp overexpressing chemoresistant cells and induces apoptosis and autophagy.

### Apoptosis and autophagy are differentially enhanced by GMI, TG and TM in MDR lung cancer sublines

**Fig 3:**
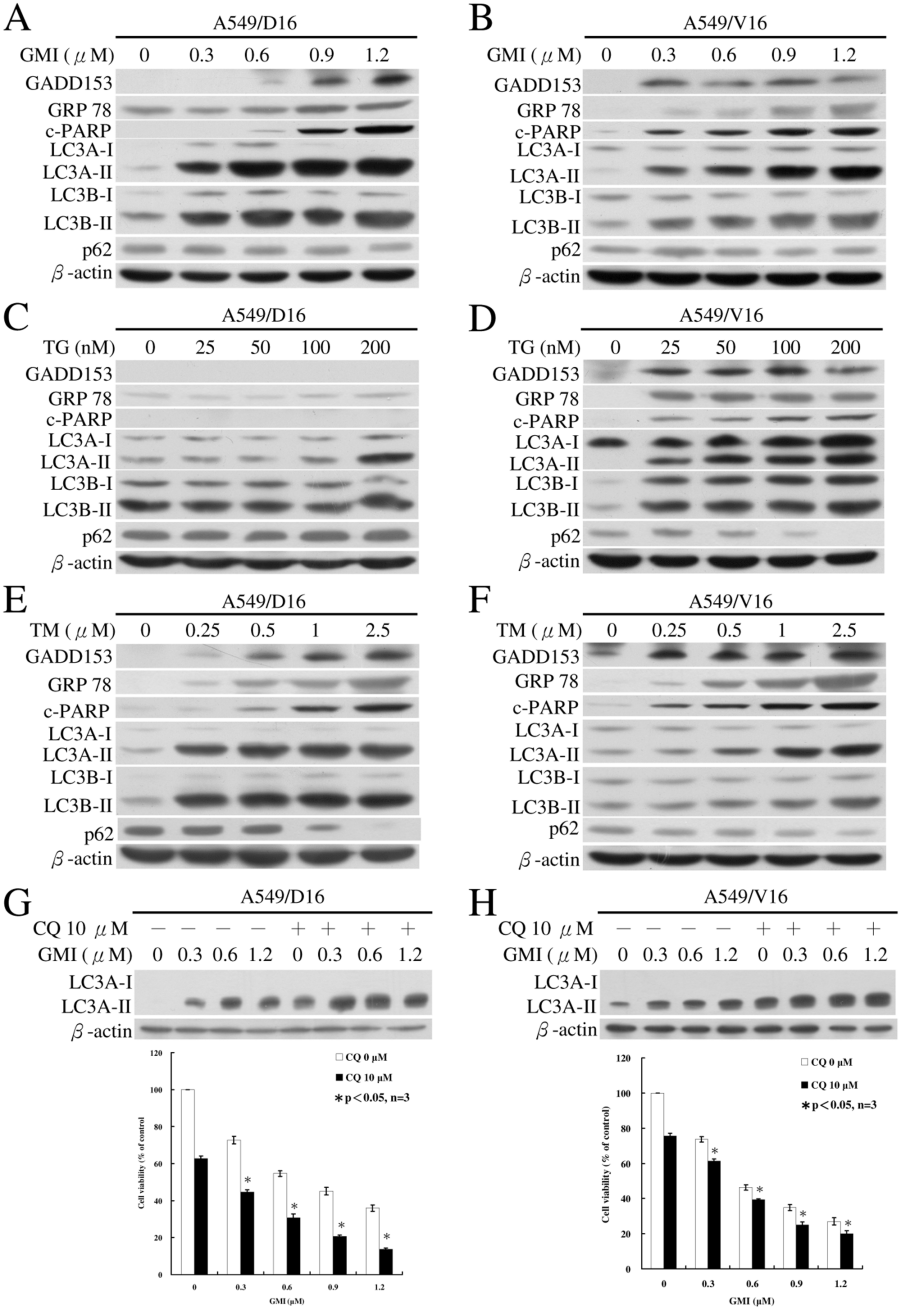
Analyses of GMI, TG and TM regulated ER stress, apoptosis and autophagy by Western blotting. (A) A549/D16 cells (2×10^5^ cells) were treated with various concentrations of GMI for 48 h. Levels of GADD153, GRP78, c-PARP, LC3A-I, LC3B-I, LC3A-II, LC3B-II and p62 were detected by Western blotting. Similar conditions were applied to (B) A549/V16 cells treated with GMI. (C) Western blot analysis of the protein levels in A549/D16 subline and (D) A549/V16 subline induced by TG. (E) A549/D16 cells were treated with various concentrations of TM for 48 h. Similar conditions were applied to (F) A549/V16 cells. (G and H) Both sublines were treated with indicated GMI concentration with the absent or present of chloroquine. The expression of β-actin served as a loading control.

To determine if the effects of GMI, TG and TM on apoptosis and autophagy are dose-dependent, we treated MDR sublines with different concentrations of GMI, TG and TM followed by the characterization of the molecular markers on immunoblots. One of the components of the ER stress-mediated apoptosis pathway is C/EBP homologous protein (CHOP), also known as growth arrest- and DNA damage-inducible gene 153 (GADD153). Another ER stress mediated protein is GRP78, also known as Bip [11]. ER stress-related GADD153 and GRP78 proteins were detected in the A549/D16 (Fig 3A) and A549/V16 (Fig 3B) sublines following GMI treatment. The apoptosis-mediated protein c-PARP was upregulated following GMI treatment as were the autophagic markers LC3A-II and LC3B-II proteins.

GADD153 protein was not detected in A549/D16 subline (Fig 3C) but was induced by TG in A549/V16 subline (Fig 3D). The proteins of GRP78 could be faintly detected with longer exposure time (Fig 3C). They were found in significantly higher quantities in A549/V16 subline (Fig 3D) on immunoblot. There was a dose-dependent induction of GADD153 by TM in the A549/D16 (Fig 3E) and the A549/V16 (Fig 3F) sublines. The levels of c-PARP proteins coordinated well with GADD153 protein levels in both sublines (Fig 3D, 3E, and 3F). Our data also showed that LC3A-II is expressed in the A549/D16 subline and increases following 200 nM TG treatment, whereas the level of LC3B-II does not increase even under high dose (200 nM) TG treatment (Fig 3C) and a longer exposure time on immunoblot. Interestingly, under TBA selection, both A549/D16 and A549/V16 sublines showed low levels of LC3B-II expression (Fig 3A, 3C, 3E, 3B, 3D and 3F, 0 nM, respectively). TG and TM enhanced the accumulation of LC3-II proteins in a dose dependent manner in A549/V16 MDR subline but not in A549/D16 subline, which expressed high level of P-gp following TG treatment (Fig 3C). Interestingly, when p62, the autophagy flux indicator that is degraded in autolysosomes [33], was monitored in MDR sublines following GMI treatment, its expression was not reduced when LC3-II increased (Fig 3A and 3B). In contrast, the protein levels of p62 diminished in TG (Fig 3D) and TM-treated sublines (Fig 3E and 3F). The formation of LC3-II and significant breakdown of p62 indicate the initiation and completion of autophagic flux following TG and TM treatment in MDR sublines (Fig 3D, 3E and 3F). In our previous report, we demonstrated that GMI-induced autophagosome accumulation results in autophagic death in A549 cells [31]. This may explain the steady level of p62 in GMI-treated sublines (Fig 3A and 3B). We further tested whether the GMI-induced autophagic flux can be observed with a lysosomal inhibitor, chloroquine which inhibited the endogenous LC3-II turnover [24]. When compared with GMI treatment, significant accumulation of LC3A-II proteins in sublines that co-treated with chloroquine were demonstrated (Fig 3G and 3H). These data demonstrated that GMI induced autophagic flux in MDR cancer cells. From these results, augmented ER stress promotes the expressions of autophagy and apoptosis regulated proteins in MDR sublines. The data indicated that the degrees of apoptosis and autophagy are dependent on GMI, TG and TM concentrations. GMI induces ER stress, apoptosis and autophagy in MDR sublines similar to TG and TM activities but with different efficacy.

### TM and TG induce the death of MDR lung cancer sublines but TG efficacy is restricted by P-gp overexpression

**Fig 4:**
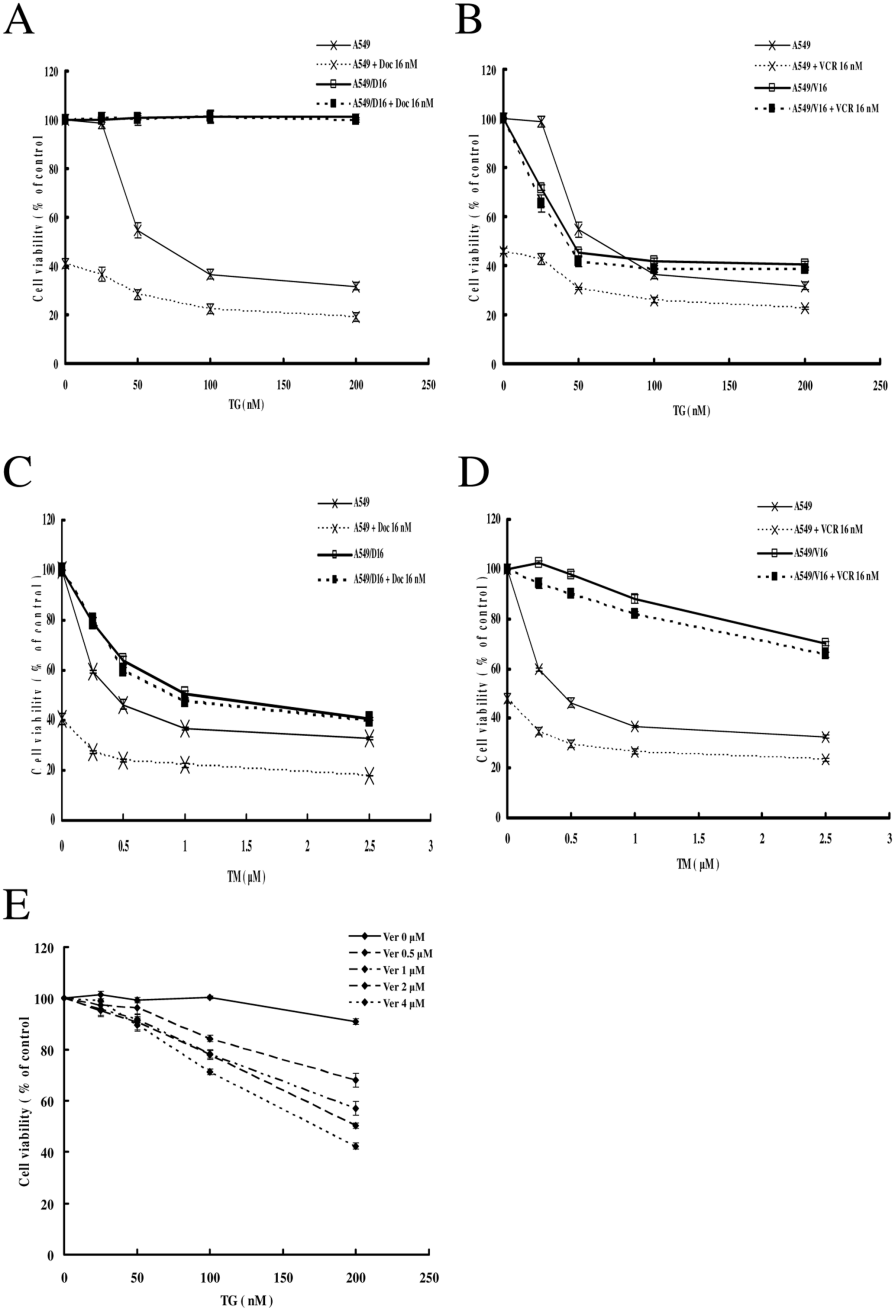
Effects of TG and TM on cell viability in MDR lung cancer cells. (A) Parental A549 and A549/D16 cells (2×10^4^ cells/well) were treated with various concentrations of TG with DOC (16 nM) or without DOC for 48 h for drug sensitivity measurements. Similar conditions were applied to (B) A549 and A549/V16 cells with VCR (16 nM). (C) Parental A549 and A549/D16 cells were treated with various concentrations of TM with DOC or without DOC for 48 h for drug sensitivity measurements. Similar conditions were applied to (D) A549 and A549/V16 cells. (E) A549/D16 cells were pre-treated with VER (0, 0.5, 1, 2 and 4 μM) for 2 h followed by various concentrations of TG for 48 h. Cell viability was analyzed on MTT assay.

We further examined the regulation of the growth of both MDR sublines by TG and TM on MTT assay. For parental A549 cells, there was approximately 32% cell survival following treatment with 200 nM TG. The cytotoxicity was enhanced when the A549 cells were cotreated with TG and DOC (16 nM), resulting in 19% cell survival at 200 nM TG. The viability of the A549/D16 subline was not affected by TG or TG cotreatment with DOC (Fig 4A). In contrast, when the A549/V16 cells were treated with TG (200 nM), 41% of cells survived (Fig 4B). Combination of VCR (16 nM) and TG had no additional cytotoxic effect on the A549/V16 subline. It has been reported that TG is a substrate of P-gp [29]. Therefore, we applied P-gp inhibitor, VER, to determine if cytotoxicity of TG can be recovered in A549/D16 subline. The results showed that when 4 μM VER was added, the viability of A549/D16 subline was reduced to 42% at 200 nM TG (Fig 4E).

The parental A549 cells were also sensitive to TM cytotoxicity in a dose-dependent manner. When the A549 cells were treated with TM (2.5 μM), approximately 33% survived. When DOC was combined with TM, 18% of the parental A549 cells remained. The A549/D16 subline also responded to TM cytotoxicity but with lower sensitivity than parental cells. DOC cotreatment with TM showed no additional cytotoxicity in A549/D16 cells (Fig 4C). The A549/V16 subline was less sensitive to TM than the A549/D16 subline with 70% surviving cells at 2.5 μM TM. Cotreatment with VCR had no major effect on the viability of A549/V16 cells (Fig 4D). These data demonstrated that parental A549 cells are sensitized to TG/TM with additional cytotoxicities when DOC or VCR is combined with TG or TM. However, A549/D16 and A549/V16 sublines are less sensitive to TM than parental cells. The drug sensitivities of A549, A549/D16 and A549/V16 cells to TG and TM with calculated IC_50_ are listed in Table 1. From analysis of the data, only the A549/V16 cells were more sensitive to TG than parental A549 cells (Fig 4B). The MDR sublines were insensitive to TG (Fig 4A) and less sensitive to TM when compared with parental A549 cells (Fig 4C and 4D).

### Quantification of TG- and TM-induced apoptosis and autophagy

**Fig 5:**
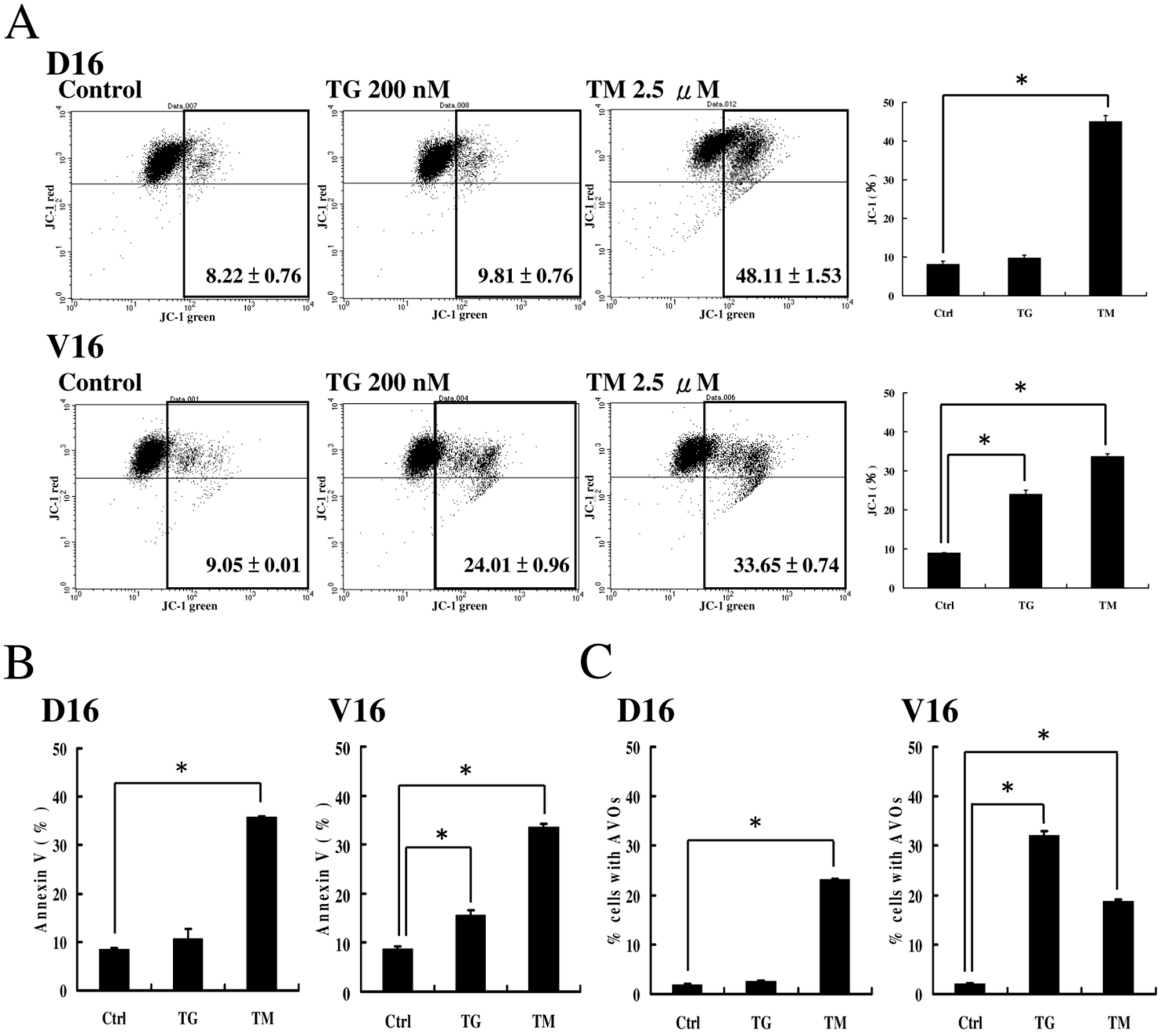
Analysis of apoptosis and autophagy by Annexin V and AVO formation in MDR sublines treated with TG and TM. (A) JC-1 assay for detection of the mitochondrial membrane potential at 24 h of TG or TM treatment in both sublines. (B) Annexin V was used to label apoptosis in untreated (Ctrl), TG- (200 nM) and TM (2.5 μM)-treated A549/D16 and A549/V16 cells for 48 h. (C) Acridine orange was used to stain AVOs in untreated (Ctrl), TG- (200 nM) and TM (2.5 μM)-treated A549/D16 and A549/V16 cells for 48 h. The cells were visualized using flow cytometry.

In addition to examining the protein markers of apoptosis, we used JC-1 and Annexin-V assay to quantify the apoptosis in TG and TM-treated sublines. When the A549/D16 subline was treated with TM for 24 h, significant apoptosis was detected by JC-1 assay but not the cells that treated with TG (Fig 5A, D16). When the A549/V16 subline was treated with same conditions, significant apoptosis was induced by TG and TM (Fig 5A, V16). When the A549/D16 subline was treated with TG for 48 h, the percentage of apoptosis was 13.58% detected by Annexin V assay. When the same subline was treated with TM for 48 h, the percentage of apoptosis was 35.86% (Fig 5B, D16). In the A549/V16 subline, the percentages of apoptosis induced by TG and TM were 15.67% and 33.55%, respectively (Fig 5B, V16). The representative dot plots for Annexin V/PI staining are showed in S1 Fig. Apoptosis is significantly induced in TM-treated sublines, indicating that TM is a better apoptosis inducer than TG.

The acridine orange of acidotropic dyes can stain late autophagic vacuoles, but not initial autophagic vacuoles, the early autophagosomes [33]. To determine the completion of autophagic flux in TG and TM-treated sublines, the formation of acidic vesicular organelles (AVOs) was characterized. The results showed that TM but not TG induces AVO development in A549/D16 subline (Fig 5C, D16). The insensitivity to TG in A549/D16 subline corresponded to P-gp overexpression, as shown in Fig 4A. Interestingly, TG triggered significant level of autophagy in A549/V16 subline and TM triggered less autophagy when compared with TG (Fig 5C, V16). From these results, TG- and TM-induced ER stresses differentially upregulate autophagy and apoptosis in MDR sublines.

### Inhibition of caspase activities represses TG/TM-induced apoptosis in VCR-resistant A549 lung cancer cells

**Fig 6:**
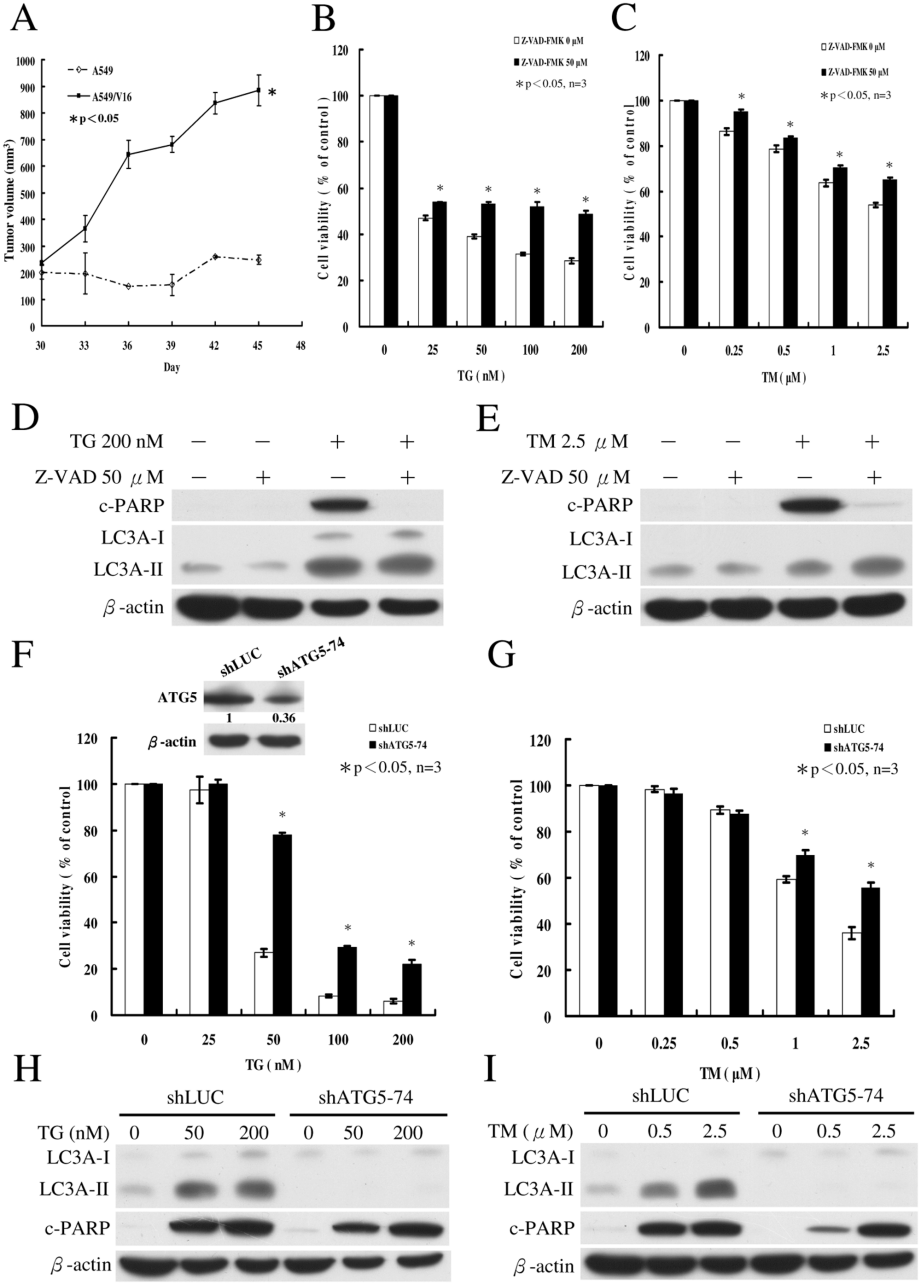
Effects of Z-VAD-FMK and ATG5 silencing on ER stress-induced cell death of A549/V16 subline. (A) A549 and A549/V16 cells (5 x 10^6^ cells in 100 μl) plus 100 μl Matrigel (BD Biosciences, 354234) were injected into the flank of each NOD SCID mouse (n = 3) and tumor volumes were monitored for 45 days. When tumor volume reached 200 mm^3^ at 30 days after implantation, the animals were intraperitoneally injected with 200 μl vinblastine sulfate (4.8 mg/kg, Vinblastine Teva, Netherlands) on days 30 and 37. The results are presented as mean ± SD. Mann-Whitney U test was applied with p<0.05 was considered statistically significant. (B) A549/V16 cells were pretreated with pan-caspase inhibitor Z-VAD-FMK (50 μM) for 1 h followed by exposure to various concentrations of TG for 48 h. Similar conditions were applied to (C) cells treated with various concentrations of TM. Cell viability was measured on MTT assay. (D) Western blot analyses of c-PARP and LC3A levels in cells incubated for 48 h in the absence (-) or presence (+) of TG and Z-VAD-FMK. Similar conditions were applied to (E) cells treated with TM. (F) The shLuc and shATG5-74 of A549/V16 cells were treated with various concentrations of TG for 48 h. Similar conditions were applied to (G) cells treated with various concentrations of TM. ATG5 expressions in cells were determined by immunoblotting with anti-ATG5 antibody. (H) Western blot analysis of proteins in cells incubated for 48 h with TG (0, 50 and 200 nM). Similar conditions were applied to (I) cells treated with TM (0, 0.5 and 2.5 μM). Asterisk (*) indicates *P*<0.05 on Student’s *t*-test, which was considered significant.

Due to the insensitivity of A549/D16 subline to TG, we used A549/V16 subline to further investigate the roles of apoptosis and autophagy in the death of MDR subline. To confirm whether A549/V16 subline is truly regulated by chemoresistance, we first tested VCR-resistance in mice xenograft model (Fig 6A). The tumor volume of A549/V16 subline continuously increased, even after injection of vinblastine sulfate. However, the tumor volume of parental A549 cells did not increase under the same conditions. To investigate the contribution of apoptosis to TG/TM-mediated death of the A549/V16 subline, cells were pretreated with pan-caspase inhibitor Z-VAD-FMK followed by TG or TM. As shown in Fig 6B, the percentage of surviving cells was reduced to 28% at a concentration of 200 nM TG on MTT assay. Inhibition of caspases resulted in significant enhancement of the cell survival to 48%. Under similar conditions, addition of TM (2.5 μM) reduced cell survival to 54% and caspase inhibition increased cell survival to 65% (Fig 6C). The inhibition of caspase activities was further demonstrated by immunoblotting of c-PARP. When Z-VAD-FMK was applied, c-PARP was not produced in cells treated with TG (Fig 6D) or TM (Fig 6E). Inhibition of caspases did not significantly alter the TG (Fig 6D) or TM-induced LC3A-II expressions (Fig 6E). The data suggested that inhibition of apoptosis only partially prevents TG/TM-mediated cell death in MDR sublines. It has been discussed that under certain conditions, apoptosis and autophagy are two independent processes [32]. However, apoptosis is sometimes inhibited by the activation of autophagy [34,35] or autophagy occurs upstream of apoptosis [32]. Thus, it is essential to determine the role of autophagy in TG/TM-mediated death of MDR lung cancer cells.

### Repression of ATG5 expression reduces autophagy and cell death without altering apoptosis in TG- and TM-treated A549/V16 cells

The ATG5 protein is required for autophagosome formation [36]. Thus, ATG5 gene silencing experiments were carried out with VZV-G pseudotyped lentivirus-shRNA system. When the expression of ATG5 was knockdowned via shRNA, there were reductions in the ATG5 protein level in the shATG5-74 clone (Fig 6F, inserted panel) and the cytotoxicities of TG (Fig 6F) and TM (Fig 6G) in A549/V16 shATG5-74 cells. The insensitivity of shATG5-74 clone to TG at a concentration of 25 nM may have been due to antibiotic selection of the recombinant lentiviruses in which TG sensitivity was altered. Treatment of mock-controlled shLUC of A549/V16 cells with TG (50 nM) resulted in 27% cell viability. However, the shATG5-74 cells maintained 78% viability under the same conditions. At a 200 nM concentration of TG, only 6% of shLUC-A549/V16 cells survived, in contrast to 22% of shATG5-74 cells (Fig 6F). Treatment with TM (2.5 μM) resulted in 36% viability of shLUC-A549/V16 cells. Reduced ATG5 expression increased viability to 55% (Fig 6G). These results indicated that reduced autophagy by ATG5 knockdown provides protection to MDR cells from TG and TM. In A549/V16 shATG5-74 cells, TG- (Fig 6H) and TM-induced LC3A-II conversions were blocked (Fig 6I), but LC3A-II levels accumulated in control cells (shLUC-A549/V16) treated with TG and TM. This further demonstrated that ATG5 knockdown represses autophagosome development. Surprisingly, the c-PARP protein levels of shATG5-74 cells were not significantly affected by ATG5 inhibition. This indicated that apoptosis and autophagy are two independent processes in TG/TM-mediated death of A549/V16 subline.

### GMI-induced autophagy plays a pro-apoptotic role in the death of MDR lung cancer sublines

**Fig 7:**
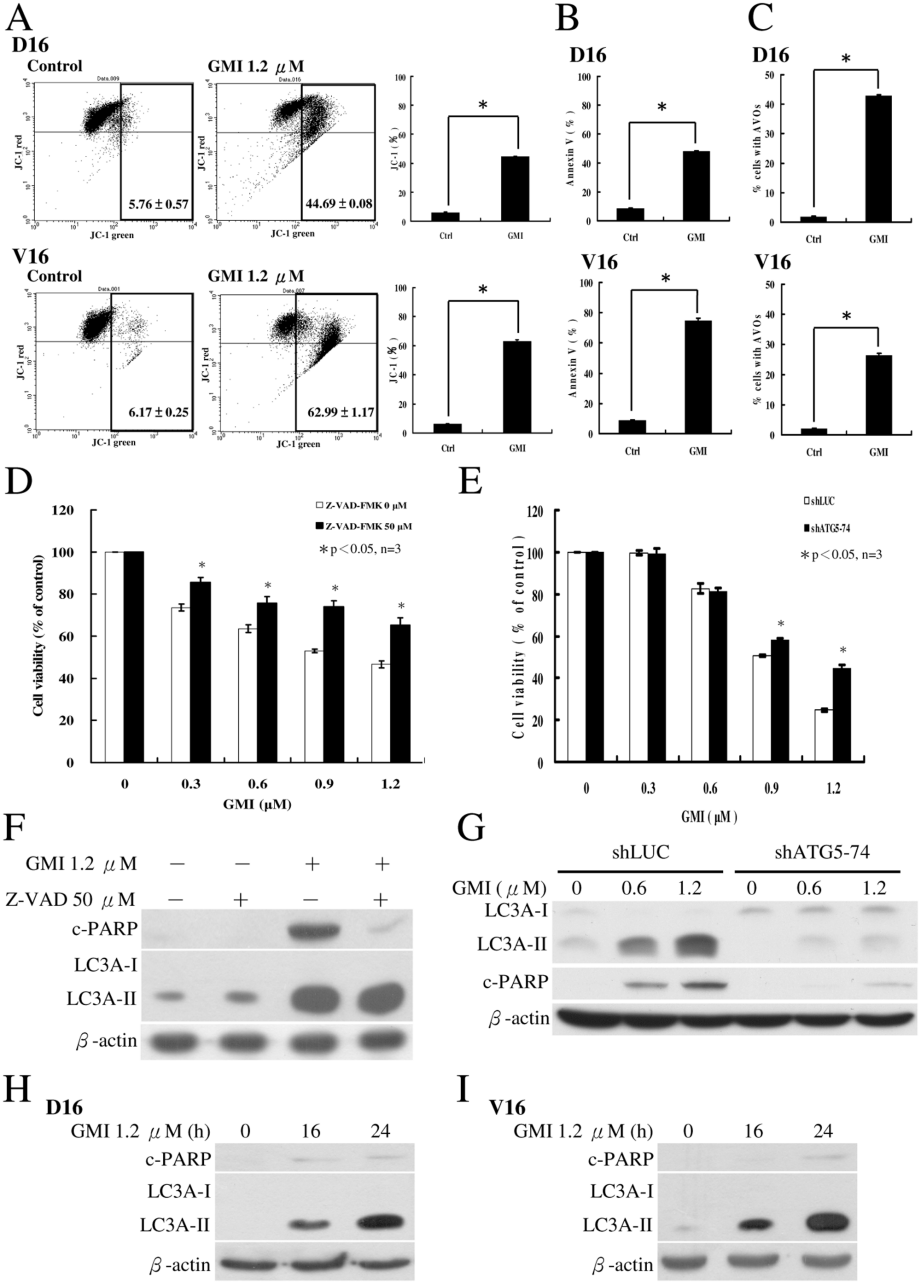
Characterization of the effects of GMI in MDR lung cancer sublines. (A) JC-1 assay to measure the mitochondrial membrane potential at 24 h of GMI treatment in both sublines. (B) Analysis of apoptosis on Annexin V assay (C) and autophagy by AVO formation in cells treated with GMI (1.2 μM). (D) A549/V16 cells were pretreated with pan-caspase inhibitor Z-VAD-FMK (50 μM) for 1 h followed by exposure to various concentrations of GMI for 48 h and analysis on MTT assay. (F) Protein levels in cells were analyzed on Western blot (E) shLuc and shATG5-74 of A549/V16 cells were treated with various concentrations of GMI for 48 h then analyzed on MTT assay. (G) Protein levels in cells were analyzed on Western blot. (H) Detection of the LC3A-II and c-PARP occurrence at 16 h and 24 h of GMI treatment in A549/D16 subline and (I) A549/V16 subline.

Although TG and TM can induce the death of MDR lung cancer cells, the high cytotoxicity of these two drugs eliminates them as viable treatment options for cancer patients. To determine whether GMI-induced apoptosis and autophagy are two independent processes that circumvent MDR, we further characterized the GMI activity with MDR sublines *in vitro*. Both sublines were treated with GMI for 24 h and significant apoptosis were detected by JV-1 assay (Fig 7A). The percentages of apoptosis on Annexin V assay were 48.07% and 74.67% in the A549/D16 and A549/V16 sublines, respectively (Fig 7B and S1 Fig). The percentages of autophagic AVOs were 42.9% and 26% in the A549/D16 and A549/V16 sublines, respectively (Fig 7C). To investigate the contribution of apoptosis to the GMI-mediated death of the A549/V16 subline, cells were pretreated with pan-caspase inhibitor Z-VAD-FMK followed by GMI. Inhibition of caspases resulted in higher percentage of surviving cells (Fig 7D). The c-PARP diminished when caspases were inhibited but there was no effect on LC3A-II levels (Fig 7F). When the ATG5 expression was interfered with by shRNA in shATG-74 clone, viability of cells increased (Fig 7E) following GMI treatment. Interestingly, when autophagic responses were repressed in shATG-74 clone, c-PARP protein levels were reduced (Fig 7G). Furthermore, we also investigated the occurrence of LC3A-II in comparison with c-PARP (Fig 7H and 7I). The level of LC3A-II increased significantly at 16 h of GMI treatment when only negligible c-PARP was detected in both sublines. This suggests that high level of autophagy leads to apoptosis following GMI treatment. However, this is not similar to TG/TM-mediated death of MDR sublines in that apoptosis is not affected by autophagy. To rule out the possibility of high P-gp expression restricting GMI efficacy, we used VER (0.5 to 4 μM) to inhibit activity of P-gp in A549/D16 subline. The results showed that P-gp inhibition has no significant effect on GMI efficacy (data not shown). Thus, the cytotoxicity of GMI was not blocked by high P-gp activity in A549/D16 subline and the results obtained from the mice xenograft model were supported (Fig 2).

### Inhibition of Akt and p70S6K phosphorylation by GMI induces autophagy independent of ERK phosphorylation, whereas Akt, p70S6K and ERK phosphorylation are all downregulated by TG and TM in MDR sublines

**Fig 8:**
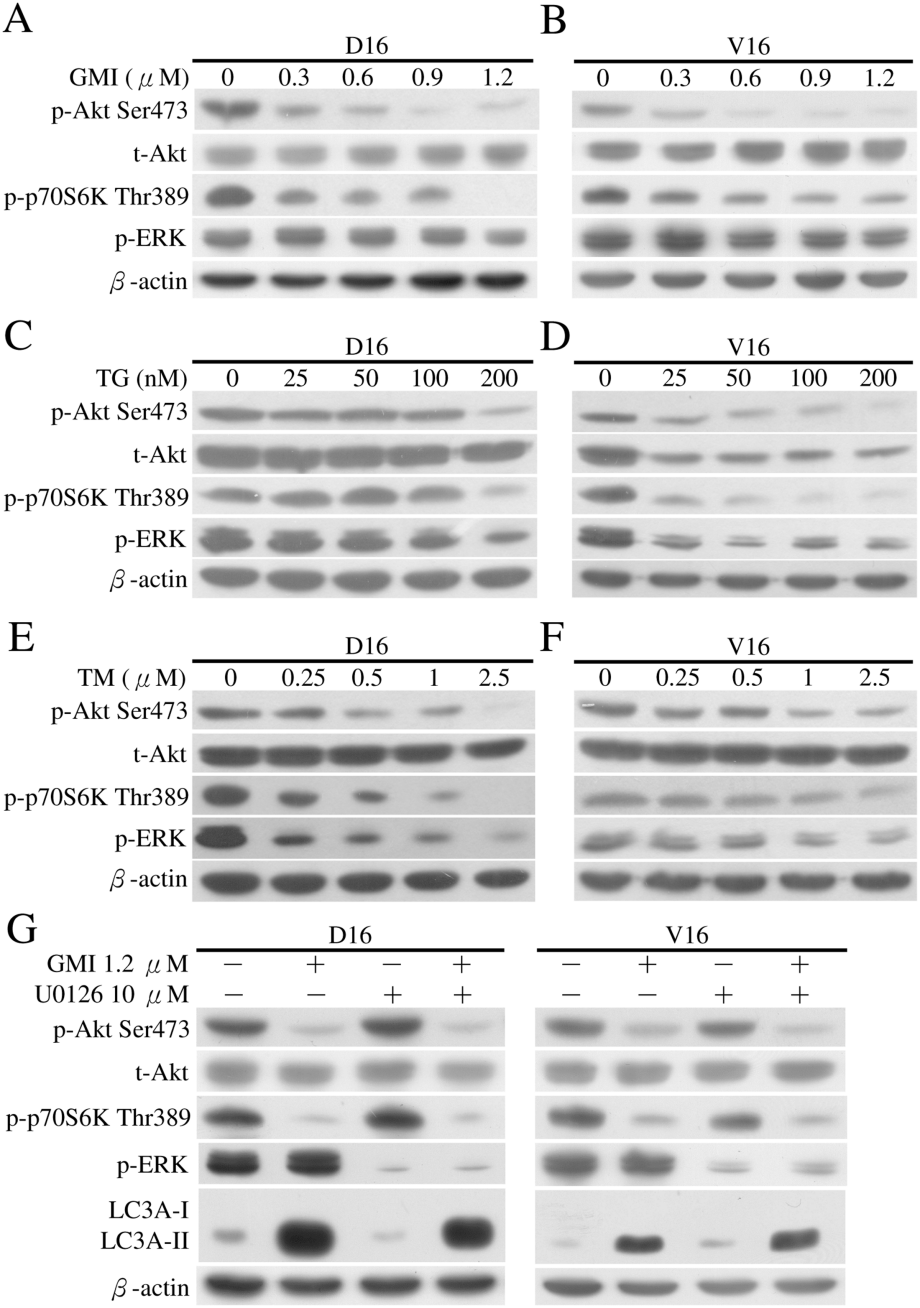
Analyses of Akt, p70S6K and ERK phosphorylation following GMI, TG and TM treatment in MDR sublines. A549/D16 and (B) A549/V16 sublines were treated with GMI (0.3–1.2 μM) for 48 h and harvested. Equal amounts of total cell lysates were analyzed on Western blot. (C) A549/D16 and (D) A549/V16 sublines were treated with TG (25–200 nM) for 48 h and harvested. (E) A549/D16 and (F) A549/V16 sublines were treated with TM (0.25–2.5 μM) for 48 h and harvested. Protein levels of total Akt (t-Akt), phosphorylated-Akt Ser473 (p-Akt Ser473), phosphorylated-p-70S6K Thr389 (p-p-70S6K Thr389) and phosphorylated-ERK (p-ERK) were determined using the corresponding antibodies. (G) The association of ERK with GMI-induced autophagy was investigated. MDR sublines were pre-treated with U0126 (10 μM) for 1 h followed by GMI (1.2 μM) for 48 h and analysis by Western blotting.

There are at least two signaling pathways that regulate autophagy. When class I phosphatidylinositol 3-phosphate kinase (PI3K)/Akt/mTOR/p70 ribosomal protein S6 kinase (p70S6K) signaling pathway is activated, autophagy is inhibited [37]. When extracellular signal-regulated kinase 1/2 (ERK1/2) pathway is activated, autophagy is induced [38]. To identify the pathway involved in GMI-induced autophagy in MDR sublines, we treated the MDR sublines with increased doses of GMI for 48 h, followed by analysis of protein lysates on Western blot (Fig 8A and 8B). The Akt-Ser473 and p70S6K-Thr389 phosphorylated levels were significantly reduced with the lowest level detected in cells treated with 1.2 μM GMI in both sublines. The phosphorylated ERK (p-ERK) levels were not upregulated under the same GMI treatment conditions. We further examined the regulation of PI3K/Akt/mTOR/p70S6K and ERK signaling pathways with TG or TM treatment in MDR sublines. Phosphorylation of Akt-Ser473 and p70S6K-Thr389 was inhibited in A549/V16 cells treated with TG (Fig 8D), A549/D16 cells treated with TM (Fig 8E) and A549/V16 cells treated with TM (Fig 8F), respectively. Only when treated with 200 nM TG did TG-resistant A549/D16 cells exhibit reduction in phosphorylated Akt-Ser473 and p70S6K-Thr389 levels (Fig 8C). In contrast to a previous report that TG and TM increase the phosphorylation of ERK in L6 muscle cells [39], ERK phosphorylation was downregulated in MDR sublines treated with TG or TM.

To rule out the possible saturation effect of p-ERK in the stable p-ERK levels, MDR sublines were pretreated with inhibitor of ERK (U0126) for 1 h followed by GMI (Fig 8G). In cells treated with U0126, there was significant inhibition of p-ERK levels with no effect on LC3A-II accumulation. Under co-treatment with GMI, phosphorylation of Akt and p70S6K remained downregulated with high levels of LC3A-II turnover in ERK-inhibited cells. Thus, GMI-induced autophagy is regulated by inhibition of Akt/mTOR/ p70S6K signaling pathway but not by ERK activation.

## Discussion

MDR sublines are sensitive to ER stressors TG and TM to differing degrees, resulting in apoptotic and autophagic cell death. In contrast to the pro-death effects in MDR sublines, TG/TM-induced autophagy has been reported to protect against cell death in HCT116 colon cancer cells and DU145 prostate cancer cells [15]. Therefore, whether cell or tumor specific autophagy under ER stress conditions is pro-survival or pro-death requires further careful investigation. In the MDR sublines in this study, mild autophagy and low [Ca^2+^]_cyt_ enabled cancer cells to survive chemotherapy, thus conferring resistance. It is important to note that the potential pro-survival role of autophagy in MDR cells can be switched to a death promoting role by strong ER stressor that overcomes chemoresistance.

Autophagy has been proposed as a target in cancer therapy, and its disruption is a promising strategy for increasing sensitivity to therapeutic agents. Endorsements of autophagy as adjuvant therapy for cancer have been made following studies of multiple tumor models [24]. Therefore, in chemotherapy, one of the strategies for enhancing anticancer efficacy is inhibition of autophagy to ensure drug sensitivity and reduced chemoresistance in the early phase of tumorigenesis. In contrast, our data indicated that when cancer develops to later phase and is refractory to chemotherapy, induction of robust ER stress followed by apoptosis and autophagy restricts chemoresistant cancer cell growth in mouse MDR model. Therefore, whether autophagy is pro-survival or pro-death may be due to its severity or duration. It has been suggested that induction of mild and/or slow autophagy enhances cell survival while severe and/or rapid autophagy causes cancer cell death [25].

The role of the PI3K/Akt/mTOR pathway in the development of resistance to standard anticancer therapy has been a recent focus of research and the inhibitors of the PI3K/AKT/mTOR pathway that restore sensitivity of cancers that have acquired resistance to standard therapy have been discussed [40]. PI3K/Akt/mTOR pathway plays a major role in cell survival, proliferation, and angiogenesis in human cancer [41]. The inhibition of PI3K/Akt/mTOR pathway results in autophagy induction. Our data suggested that to overcome MDR, a potential autophagy inducer that specifically targets PI3K/Akt/mTOR pathway and generates robust autophagy is needed. Moreover, an ideal agent for overcoming MDR must not be a substrate of P-gp and must result in low cytotoxicity to normal cells. Interestingly, we found that MDR sublines have higher sensitivity to GMI than parental lung cancer A549 cells. This may be due to the occurrence of apoptosis after autophagy in MDR sublines, but not in parental A549 cells, treated with GMI [30,31]. GMI is not a P-gp substrate and it induces autophagy-associated apoptosis with higher efficiency than TG and TM to inhibit the growth of MDR sublines. The body weights of mice were 22±0.94g and 17±0.47g before and after treatment with vinblastine (Fig 6A) respectively. The body weights of mice were 21±1.43g and 22±0.81g before and after treatment with GMI, respectively (Fig 2B). This implied no weight loss in GMI-treated animals. Therefore, GMI has lower cytotoxicity in mice than vinblastine.

ERK activation controls various cell responses, such as proliferation, migration, differentiation and death [42]. Depending on the cell type and stimulus, ERK activity also mediates different antiproliferative events, such as apoptosis, autophagy and senescence [38]. GMI inhibits PI3K/Akt/mTOR signaling pathway and induces autophagy to promote the apoptotic death of MDR cells without ERK activation. Thus, GMI is a potential Akt/mTOR signaling pathway inhibitor that is not associated with ERK signaling pathway. Recently, it has been reported that pterostilbene inhibits AKT and JNK and activates ERK1/2 pathway to promote autophagy in A549/D16 subline [43]. Autophagy blocked by ERK inhibitor (U0126) enhances pterostilbene-triggered apoptosis which implies that ERK-regulated autophagy has anti-apoptotic effect in A549/D16 cells. Therefore, the pro-death effect of GMI-induced autophagy differs from that of pterostilbene-induced autophagy. We also demonstrated in mice xenograft tumors that GMI alone is effective in restricting MDR lung cancer growth. The inhibition of ERK signaling pathway by TG or TM in MDR sublines (Fig 8D, 8E and 8F) may reflect the anti-apoptotic activity of ERK pathway in these chemoresistant cancer cells which differs from the ERK regulated pro-death role observed in other cells [38]. Thus, treatment with TG or TM inhibits ERK activation and may result in apoptosis of MDR sublines.

Dysregulated autophagy is a hallmark of cancer cells. According to our data, there are at least three types of agents that can be applied to the remission of TBA-mediated chemoresistance. The first is the L-type calcium channel blockers that can be used together with TBA for re-sensitization to chemotherapy through upregulated [Ca^2+^]_cyt_. The second is powerful ER stress inducers such as TG and TM that trigger apoptosis and autophagic cell death. The third is a fungal protein GMI that induces autophagy-associated cell death through Akt/mTOR signaling pathway inhibition without ERK activation.

Our data indicated that autophagy stimulation should be considered in acquired chemoresistance. GMI-selective targeting of Akt/mTOR signaling pathway to induce autophagy may be beneficial in the management of advanced NSCLC. As ER stress inducers are potential anticancer agents, it is worthwhile to explore how to best manipulate the resulting autophagic upregulation to maximize anticancer efficacy. Additional work is required to understand the nature of the molecular mechanisms for overcoming chemoresistance.

## Supporting Information

S1 Fig Annexin V assay of TG, TM and GMI treatment in A549/D16 and A549/V16 sublines. Representative dot plots of Annexin/PI staining in (A) A549/D16 and (B) A549/V16 cells that treated with TG (200 nM), TM (2.5 μM) and GMI (1.2 μM) for 48 h followed by analysis with flow cytometry.
(TIF)
