# Supplementary figures and images for: Immunomodulatory Protein from Ganoderma microsporum Induces Pro-Death Autophagy through Akt-mTOR-p70S6K Pathway Inhibition in Multidrug Resistant Lung Cancer Cells
Source: PLoS One. 2015 May 6;10(5):e0125774. doi: 10.1371/journal.pone.0125774 (PMC4422711; doi:10.1371/journal.pone.0125774)

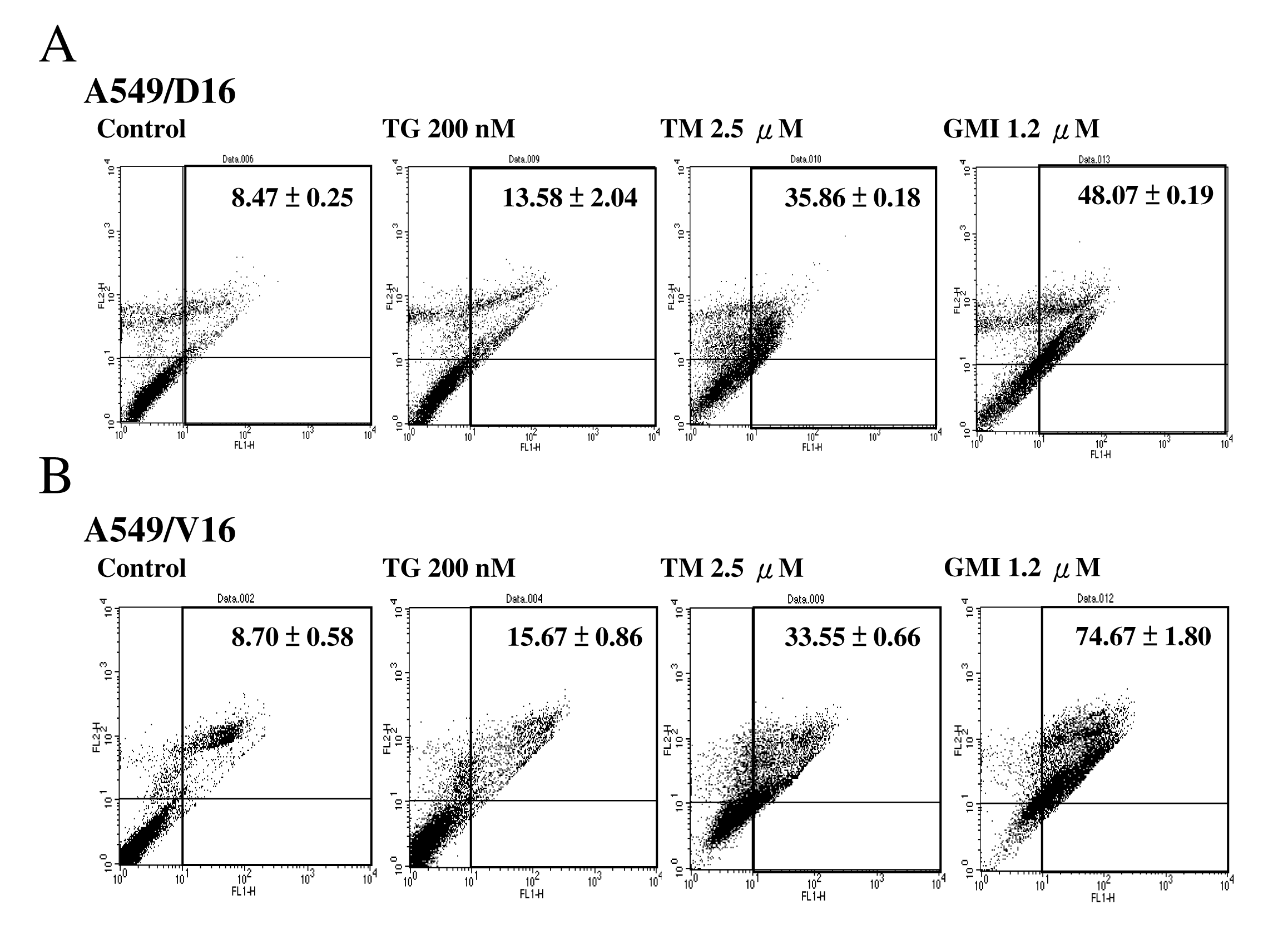

Supplement: S1 Fig — Representative dot plots of Annexin/PI staining in (A) A549/D16 and (B) A549/V16 cells that treated with TG (200 nM), TM (2.5 μM) and GMI (1.2 μM) for 48 h followed by analysis with flow cytometry. (TIF) [file pone.0125774.s001.tif]
